# Relaxin Signals through a RXFP1-pERK-nNOS-NO-cGMP-Dependent Pathway to Up-Regulate Matrix Metalloproteinases: The Additional Involvement of iNOS

**DOI:** 10.1371/journal.pone.0042714

**Published:** 2012-08-22

**Authors:** Bryna Suet Man Chow, Elaine Guo Yan Chew, Chongxin Zhao, Ross A. D. Bathgate, Tim D. Hewitson, Chrishan S. Samuel

**Affiliations:** 1 Florey Neuroscience Institutes, University of Melbourne, Parkville, Victoria, Australia; 2 Department of Biochemistry and Molecular Biology, University of Melbourne, Parkville, Victoria, Australia; 3 Department of Nephrology, Royal Melbourne Hospital, Parkville, Victoria, Australia; 4 Department of Medicine, University of Melbourne, Royal Melbourne Hospital, Parkville, Victoria, Australia; 5 Department of Pharmacology, Monash University, Clayton, Victoria, Australia; Florida International University, United States of America

## Abstract

The hormone, relaxin, inhibits aberrant myofibroblast differentiation and collagen deposition by disrupting the TGF-β1/Smad2 axis, via its cognate receptor, Relaxin Family Peptide Receptor 1 (RXFP1), extracellular signal-regulated kinase (ERK)1/2 phosphorylation (pERK) and a neuronal nitric oxide (NO) synthase (nNOS)-NO-cyclic guanosine monophosphate (cGMP)-dependent pathway. However, the signalling pathways involved in its additional ability to increase matrix metalloproteinase (MMP) expression and activity remain unknown. This study investigated the extent to which the NO pathway was involved in human gene-2 (H2) relaxin's ability to positively regulate MMP-1 and its rodent orthologue, MMP-13, MMP-2 and MMP-9 (the main collagen-degrading MMPs) in TGF-β1-stimulated human dermal fibroblasts and primary renal myofibroblasts isolated from injured rats; by gelatin zymography (media) and Western blotting (cell layer). H2 relaxin (10–100 ng/ml) significantly increased MMP-1 (by ∼50%), MMP-2 (by ∼80%) and MMP-9 (by ∼80%) in TGF-β1-stimulated human dermal fibroblasts; and MMP-13 (by ∼90%), MMP-2 (by ∼130%) and MMP-9 (by ∼115%) in rat renal myofibroblasts (all p<0.01 vs untreated cells) over 72 hours. The relaxin-induced up-regulation of these MMPs, however, was significantly blocked by a non-selective NOS inhibitor (L-nitroarginine methyl ester (hydrochloride); L-NAME; 75–100 µM), and specific inhibitors to nNOS (N-propyl-L-arginine; NPLA; 0.2–2 µM), iNOS (1400W; 0.5–1 µM) and guanylyl cyclase (ODQ; 5 µM) (all p<0.05 vs H2 relaxin alone), but not eNOS (L-N-(1-iminoethyl)ornithine dihydrochloride; L-NIO; 0.5–5 µM). However, neither of these inhibitors affected basal MMP expression at the concentrations used. Furthermore, of the NOS isoforms expressed in renal myofibroblasts (nNOS and iNOS), H2 relaxin only stimulated nNOS expression, which in turn, was blocked by the ERK1/2 inhibitor (PD98059; 1 µM). These findings demonstrated that H2 relaxin signals through a RXFP1-pERK-nNOS-NO-cGMP-dependent pathway to mediate its anti-fibrotic actions, and additionally signals through iNOS to up-regulate MMPs; the latter being suppressed by TGF-β1 in myofibroblasts, but released upon H2 relaxin-induced inhibition of the TGF-β1/Smad2 axis.

## Introduction

Fibrosis is a universal response to chronic injury and inflammation in several organs and its failure to resolve leads to significant dysfunction and onset of organ failure [1], [2]. Under pathological conditions, excessive collagen deposition (the main constituent of fibrotic tissue) leads to adverse outcomes, with damage depending not only on the quantity of matrix produced (fibrogenesis), but also the degree of its cross-linking and its reorganisation, or density.

Fibrosis is dependent to a large extent on the recruitment of myofibroblasts, cells with the phenotypic features of both fibroblasts and vascular smooth muscle [3]. Recognised by their *de novo* expression of α smooth muscle actin (αSMA), myofibroblasts are prodigious producers of the ECM and are influenced by several mediators, including cytokines, chemokines and growth factors [3], [4]. A hierarchy of these is likely to exist, with transforming growth factor (TGF)-β1 amongst the most important. Expression of TGF-β1 can be induced by mechanical overload, myocardial ischemia, cardiomyopathy or angiotensin II (Ang II) [5], [6].

Finally, newly secreted matrix is remodelled and reorganised. Matrix metalloproteinases (MMP) are a family of proteinases that degrade collagens and therefore contribute to tissue remodelling [6], [7]. The activity of MMPs can be regulated i) at the transcription level, ii) through activation of latent pro-MMPs, and iii) by inhibition by tissue inhibitors of MMPs (TIMPs) that directly bind to and inhibit activated MMPs [7]. Basal expression and activity of MMPs are very low but increased significantly under diseased conditions. An imbalance of matrix synthesis/degradation can result in fibrosis or alternatively, excessive collagen degradation.

The naturally occurring hormone, relaxin, is increasingly being recognised for its ability to abrogate fibrosis in several organs and prevent and/or reverse aberrant collagen deposition in numerous experimental models of disease, regardless of etiology (reviewed in [8]–[13]). Furthermore, its other pleiotropic actions, including its vasodilatory [14], [15], angiogenic [11], [14], [16] and anti-apoptotic [17]–[19] effects are thought to facilitate organ protection and wound healing. The anti-fibrotic actions of relaxin are mediated through its cognate G-protein coupled receptor, Relaxin Family Peptide Receptor 1 (RXFP1) [20] and its ability to directly inhibit TGF-β1 signal transduction/activity [18], [20]–[22]. This in turn, limits the influence of TGF-β1-on myofibroblast differentiation, and the subsequent ability of these cells to synthesize various matrix proteins, such as collagen and fibronectin [18], [20]–[27]. Furthermore, relaxin has additionally been found to augment MMP-induced matrix degradation in a number of organs, while inhibiting the actions of TIMPs; or at least favouring a net increase in the MMP:TIMP ratio [18], [19], [21]–[30].

The signal transduction pathways by which relaxin mediates its anti-fibrotic actions are still to be fully understood, but are key to identifying novel targets that may be used to enhance its therapeutic potential. To date, studies from human [21] and rodent [20], [23] renal myofibroblasts have demonstrated that relaxin acts through RXFP1, extracellular signal-regulated kinase (ERK)1/2 phosphorylation (pERK) and a neuronal nitric oxide (NO) synthase (nNOS)-NO-cyclic guanosine monophosphate (cGMP)-dependent pathway to inhibit the phosphorylation of Smad2 (a regulatory protein that promotes TGF-β1 activity and signalling); as a means of disrupting the actions of TGF-β1. The ability of relaxin to inhibit Smad2 phosphorylation (pSmad2) and TGF-β1-induced myofibroblast differentiation and collagen production has subsequently been demonstrated in an experimental model of tubulointerstitial fibrosis *in vivo* [18], and in rat ventricular fibroblasts *in vitro* [22]; suggesting that its ability to abrogate the TGF-β1/Smad2 axis are both species and organ independent. However, the pathways involved in its additional ability to increase MMP activity remain unknown.

On the basis of these findings, the current study aimed to further elucidate the signalling mechanisms by which relaxin positively regulates MMP-1 (collagenase-1) and its rodent orthologue, MMP-13 (collagenase-3), MMP-2 (gelatinase A) and MMP-9 (gelatinase B); the main collagen-degrading MMPs. These studies were conducted using TGF-β1-stimulated human dermal fibroblasts and primary rat renal myofibroblasts isolated from the obstructed kidneys of injured rats, which both express RXFP1 [20], [31] and respond to relaxin. More specifically the involvement of RXFP1, pERK and the nNOS-NO-cGMP-dependent pathway (which was found to be involved in relaxin's ability to inhibit renal myofibroblast differentiation [20]); the additional involvement of iNOS and eNOS in this process; and the link between relaxin's ability to stimulate pERK and separately, nNOS expression in renal myofibroblasts [20], was further investigated.

## Methods

### Materials

Recombinant human gene-2 (H2) relaxin was generously provided by Corthera (San Mateo, CA, USA; a subsidiary of Novartis International AG, Basel, Switzerland); and is the major stored and circulating form of human relaxin, which is bioactive in rats [20], [22]–[24], [27], [30] and mice [18]–[20], [25]. BJ3 cells, a human dermal fibroblast cell line [32] was kindly provided by Dr. William C. Hahn (Department of Medical Oncology, Dana-Farber Cancer Institute, Boston, MA). Recombinant human TGF-β1 was obtained from R&D Systems (Minneapolis, MN, USA).

### Animals

Tissue and cells isolated from the obstructed kidneys of male Sprague-Dawley rats that were subjected to unilateral ureteric obstruction (UUO); and nNOS wild-type (nNOS^+/+^) and knockout (nNOS^−/−^) litter-mate mice (from heterozygous breeders) that were treated with or without H2 relaxin, were used to study relaxin signal transduction pathways. UUO is a rapid and reproducible model of primary tubulointerstitial fibrosis, occurs independently of species and strain, demonstrates changes that mimic the pathology of human progressive renal disease [33], and was performed as described before [18], [20].

The animals were housed in a controlled environment and maintained on a fixed lighting schedule with free access to rodent lab chow (Barastock Stockfeeds, Pakenham, Victoria, Australia) and water. These experiments were approved by the Florey Neuroscience Institutes' Animal Ethics Committee, which adheres to the Australian code of practice for the care and use of laboratory animals for scientific purposes.

### Propagation and culture of renal myofibroblasts

Previous studies have shown that cells propagated from fibrotic kidneys are more active than fibroblasts grown from normal kidneys [34], and are therefore a better reflection of their *in vivo* counterparts. For these studies, myofibroblasts were propagated from rat kidney tissue 3 days after UUO, as described previously [20], [23]. Cells were characterized immunocytochemically and were positive for the mesenchymal marker vimentin, negative for the epithelial marker cytokeratin, and only occasionally positive for desmin (a marker of mesangial cells and some myofibroblasts). As between 60–70% of cells stained for α-smooth muscle actin (SMA), it was concluded that fibroblasts constituted 100% of the cells used for experimentation, of which 60–70% were myofibroblasts. Cultures were maintained in DMEM supplemented with 10% fetal calf serum (FCS), penicillin (50 U/ml) and streptomycin (50 µg/ml). Rat myofibroblasts were used between passages 15 and 25 for the outlines studies; and all experiments were performed at least 3–5 separate times in duplicate.

### Culture of human dermal fibroblasts (BJ3 cells)

BJ3 cells (human dermal fibroblasts) were elongated in appearance and characterized as described above; where fibroblasts constituted the cells used for experimentation. These cells were cultured in DMEM containing 17% Medium 199, 15% FCS, penicillin (50 U/ml), streptomycin (50 µg/ml), 1% L-glutamine and 2.2% HEPES. Human dermal fibroblasts were used between passages 7 and 17 for the outlined studies, while all experiments were performed at least 3–4 separate times in duplicate.

### Determination of latent and active MMPs

To study the effects of H2 relaxin on MMP expression and activity, BJ3 cells or rat renal myofibroblasts were seeded into 12-well plates at an equal density of 1–1.25×10^5^ cells/well and maintained in their respective media (detailed above) for experimental conditions. The inclusion of FCS was necessary to ensure survival of these cell cultures. As TGF-β is both found in FCS and made in an autocrine fashion by myofibroblasts, all cells were exposed to some TGF-β. Furthermore, exogenous human recombinant TGF-β1 (2 ng/ml) was also immediately added to BJ3 cells to ensure differentiation of fibroblasts into myofibroblasts, and to study the effects of H2 relaxin on TGF-β1 signal transduction. On the other hand, renal myofibroblasts retained their phenotype up to 25 passages in culture (in the absence of exogenous TGF-β1 administration).

#### BJ3 cell studies

Based on H2 relaxin (0.1–100 ng/ml) dose-response studies (by gelatin zymography; data not shown); which showed that relaxin was able to stimulate latent and active MMP-1, MMP-2 and MMP-9 levels at all concentrations tested; with MMP-1 maximally increased with 10 ng/ml; MMP-2 maximally increased with 0.1, 1, 10 and 100 ng/ml; and MMP-9 increased in an inverse bell-shaped pattern, with maximal stimulation at 1 ng/ml and no difference in stimulation between 1 ng/ml and 10 ng/ml; 10 ng/ml (1.68 nM) H2 relaxin was used to stimulate these MMPs in human dermal fibroblasts for all described studies.

#### Rat renal myofibroblast studies

Based on previous studies which demonstrated that H2 relaxin was able to maximally stimulate MMP-13 (the rodent orthologue of MMP-1) in these cells at 100 ng/ml [23]; while this dose of the peptide significantly inhibited Smad2 phosphorylation and the subsequent effects of TGF-β1 on renal myofibroblast differentiation [20]; 100 ng/ml (16.8 nM) H2 relaxin was used to stimulate MMP-13, MMP-2 and MMP-9 in rat renal myofibroblasts for all described studies.

In each case, BJ3 cells were stimulated with TGF-β1 (2 ng/ml) and H2 relaxin (10 ng/ml), while rat renal myofibroblasts stimulated with H2 relaxin (100 ng/ml) for 72 hours. The media was then collected, assessed for total protein by the Bradford protein assay and stored for subsequent analyses of MMP-1, MMP-2 and MMP-9 by gelatin zymography; while protein was then extracted from the cell layer with Trizol reagent (0.5 ml per well; Invitrogen Corp., Carlsbad, CA, USA; according to the manufacturer's instructions) for the analysis of MMP-13 (by Western blotting).

To determine the extent to which the NO pathway was involved in H2 relaxin's ability to positively regulate MMP-1/-13, MMP-2 and MMP-9, TGF-β1 (2 ng/ml)-stimulated BJ3 cells and rat renal myofibroblasts were treated with H2 relaxin (10 ng/ml or 100 ng/ml, respectively) in the absence of presence of the non-selective NOS inhibitor, L-nitroarginine methyl ester (hydrochloride) (L-NAME; 75–100 µM; Sigma-Aldrich, St. Louis, MO, USA); and specific inhibitors to nNOS (N-propyl-L-argnine; NPLA; 0.2–2 µM; Cayman Chemical, Ann Arbour, MI, USA); inducible (i)NOS (N-(3-(aminomethyl)benzyl)acetamidine; 1400W; 0.5–1 µM; Cayman Chemical); and endothelial (e)NOS (L-N-(1-iminoethyl)ornithine dihydrochloride; L-NIO; 0.5–5 µM; Merck kGaA; Darmstadt, Germany); in addition to the guanylyl cyclase inhibitor, 1H-(1,2,4)oxadiaazolo[4,3-a]quinoxaline-L-one (ODQ; 5 µM; Sigma-Aldrich). Untreated cells or cells treated with each of these inhibitors alone (at the same concentrations detailed above) for 72 hours were used as appropriate controls.

Additional studies in rat renal myofibroblasts were conducted to determine if i) these cells expressed iNOS (as they were previously found to express nNOS, which was up-regulated by H2 relaxin (100 ng/ml) administration, but not eNOS [20]); ii) iNOS expression was altered by exogenous TGF-β1 administration, as previously demonstrated in lung myofibroblasts [35] and other cell types [36], [37], in the absence or presence of H2 relaxin; and iii) the ability of H2 relaxin to stimulate pERK in these cells was linked to its ability to promote nNOS expression, by determining if the relaxin-induced up-regulation of nNOS in these cells was blocked by the ERK1/2 inhibitor, PD98059 (1 µM; Cell Signaling Technology, Danvers, MA, USA).

### Evaluation of MMPs from nNOS-null mice

To confirm the involvement of nNOS in the H2 relaxin-induced up-regulation of MMP-2, MMP-9 and MMP-13 *in vivo*, 5–6-week old male nNOS^+/+^ and nNOS^−/−^ mice (n = 4 per genotype; kindly provided by Professor Glenn McConell, Dept. of Physiology, University of Melbourne, Parkville, Victoria, Australia; on a C57B6J background) were continuously treated with H2 relaxin (0.5 mg/kg/day; via subcutaneously implanted osmotic mini-pumps; model 1007D; Alzet, Cupertino, CA, USA) for 7 days. This was based on previous studies showing that H2 relaxin could stimulate MMPs in the absence of TGF-β1 [25], [38] and injury [39]; while this dose of H2 relaxin was found to produce ∼20 ng/ml of circulating relaxin in mice, 5 days post-administration [40]. Basal kidney MMP levels were determined from age-matched untreated nNOS^+/+^ and nNOS^−/−^ mice (n = 3 per genotype). 7 days post-H2 relaxin administration, the untreated and treated animals were killed by an overdose of anesthetic and kidney tissues isolated for the assessment of MMP-2 and MMP-9 (by gelatin zymography), in addition to MMP-13 (by Western blotting).

### Gelatin zymography

Gelatin zymography of conditioned media was performed using the method described by Woessner [41] and used previously [22], [24], [38]. In all cases, zymographs consisted of 7.5% acrylamide gels containing 1 mg/ml gelatin. Equal volumes of each sample (containing 0.035–0.05 µg of total protein for the assessment of MMP-2, 1.4–1.5 µg of total protein for the assessment of MMP-9, and 14–15 µg of total protein for the assessment of MMP-1) were used (to avoid saturation of the MMP bands); and in some cases, aliquots of media were incubated with amino-phenyl mercuric acetate (APMA; 5 mM; Sigma-Aldrich; which activates latent MMPs) for 6 hours at 37°C before being assessed. Gelatinolytic activity was indicated by clear bands and densitometry of these MMP bands was performed with a GS710 Calibrated Imaging Densitometer (Bio-Rad Laboratories, Richmond, CA, USA) and Quantity-One software (Bio-Rad). The mean ± SE density of each MMP was graphed and expressed as the relative ratio of the values in the untreated control group, which was expressed as 1. To correct for intra- and inter-sample variation, the same untreated (control) samples were run on each zymograph performed; to assess the relative differences in gelatinase expression between the various treatment groups performed.

### Western blotting

Equivalent amounts of total protein (10 µg) from the cell layer of untreated and treated samples were electrophoresed under non-reducing conditions on 10.5% acrylamide gels. Western blot analyses were performed with a monoclonal antibody to MMP-13 (IM78; 1∶1000 dilution; Merck) and a goat anti-mouse secondary antibody (1∶2000 dilution; Millipore Corp., Bedford, MA, USA). iNOS expression in rat renal myofibroblasts was assessed with a polyclonal antibody (2997S; 1∶800 dilution; Cell Signaling Technology) and goat anti-rabbit secondary antibody (1∶2500 dilution; Sigma-Aldrich), while nNOS expression was assessed with a polyclonal antibody, as described previously [20]. In each case, Western blots of the house-keeping protein, α-tubulin (monoclonal antibody; 1∶8000 dilution; Millipore Corp.) were included to demonstrate equivalent loading of the protein samples. Blots were detected using the ECL detection kit (Amersham Pharmacia Biotech; according to the manufacturer's instructions); before being quantified and corrected for intra- and inter-sample variation by densitometry, as described above.

### Statistical analysis

The results were analyzed using a One-way analysis of variance (ANOVA) and the Newman-Keuls *post hoc* test for multiple comparisons between groups; using Prism 5.0 (GraphPad Software Inc., San Diego, CA, USA). All data are expressed as the mean ± SEM, with p<0.05 considered as statistically significant.

## Results

### H2 relaxin signals through the NO pathway to positively regulate MMP expression and activity

**Figure 1:**
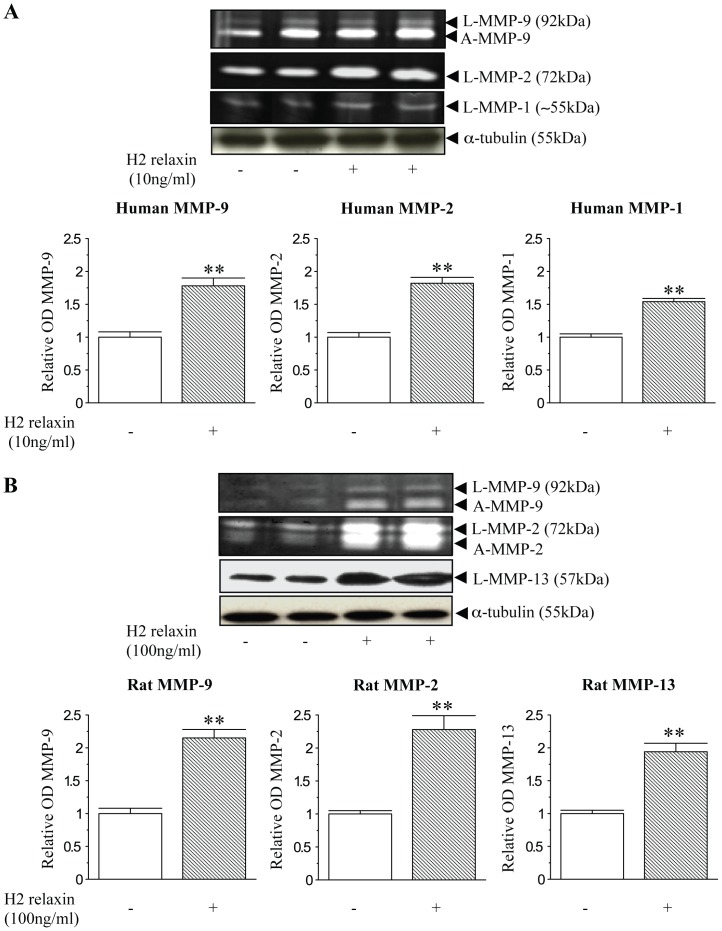
Relaxin positively regulates collagen-degrading associated MMPs in human and rat myofibroblasts. (A) Representative gelatin zymographs of latent (L) and active (A) MMP-9, L-MMP-2 and L-MMP-1 from TGF-β1-treated ± H2 relaxin (10 ng/ml)-treated human dermal fibroblasts after 72 hours. (B) Representative zymographs of L-MMP-9, A-MMP-9, L-MMP-2, A-MMP-2 and Western blots of L-MMP-13 from rat renal myofibroblasts ± H2 relaxin (100 ng/ml)-treated rat renal myofibroblasts after 72 hours. Additional blots of α-tubulin (A,B) demonstrate the quality and equivalent loading of protein samples. Media from rat renal myofibroblasts was treated with APMA (5 mM) before being assessed by zymography. Also shown are the mean ± SE levels of each human (A) and rat (B) MMP studied (which was derived from the latent and active forms), as determined by densitometry scanning (from >10 separate experiments for each cell type studied), and expressed as relative values to those of the TGF-β1 alone-treated (A) or untreated (B) groups, which was expressed as 1 in each case. **p<0.01 vs TGF-β1 alone-treated cells (A) or untreated cells (B).

Gelatin zymography and Western blot analyses identified latent and active forms of MMP-9 in addition to latent forms of MMP-2 and MMP-1 from human dermal fibroblasts (Figure 1A); in addition to latent and active forms of MMP-2 and MMP-9 as well as latent forms of MMP-13 from rat renal myofibroblasts (Figure 1B). H2 relaxin significantly increased MMP-1 (by ∼50%), MMP-2 (by ∼80%) and MMP-9 (by ∼80%) levels in TGF-β1-stimulated human dermal fibroblasts (at 10 ng/ml; all p<0.01 vs TGF-β1 alone-stimulated cells) (Figure 1A); and MMP-13 (by ∼90%), MMP-2 (by ∼130%) and MMP-9 (by ∼115%) levels in rat renal myofibroblasts (at 100 ng/ml; all p<0.01 vs untreated cells) (Figure 1B) over 72 hours in culture. During this same time, it was confirmed that H2 relaxin (100 ng/ml) significantly inhibited renal pSmad2 and α-SMA expression (myofibroblast differentiation), as demonstrated previously [20] (data not shown).

**Figure 2:**
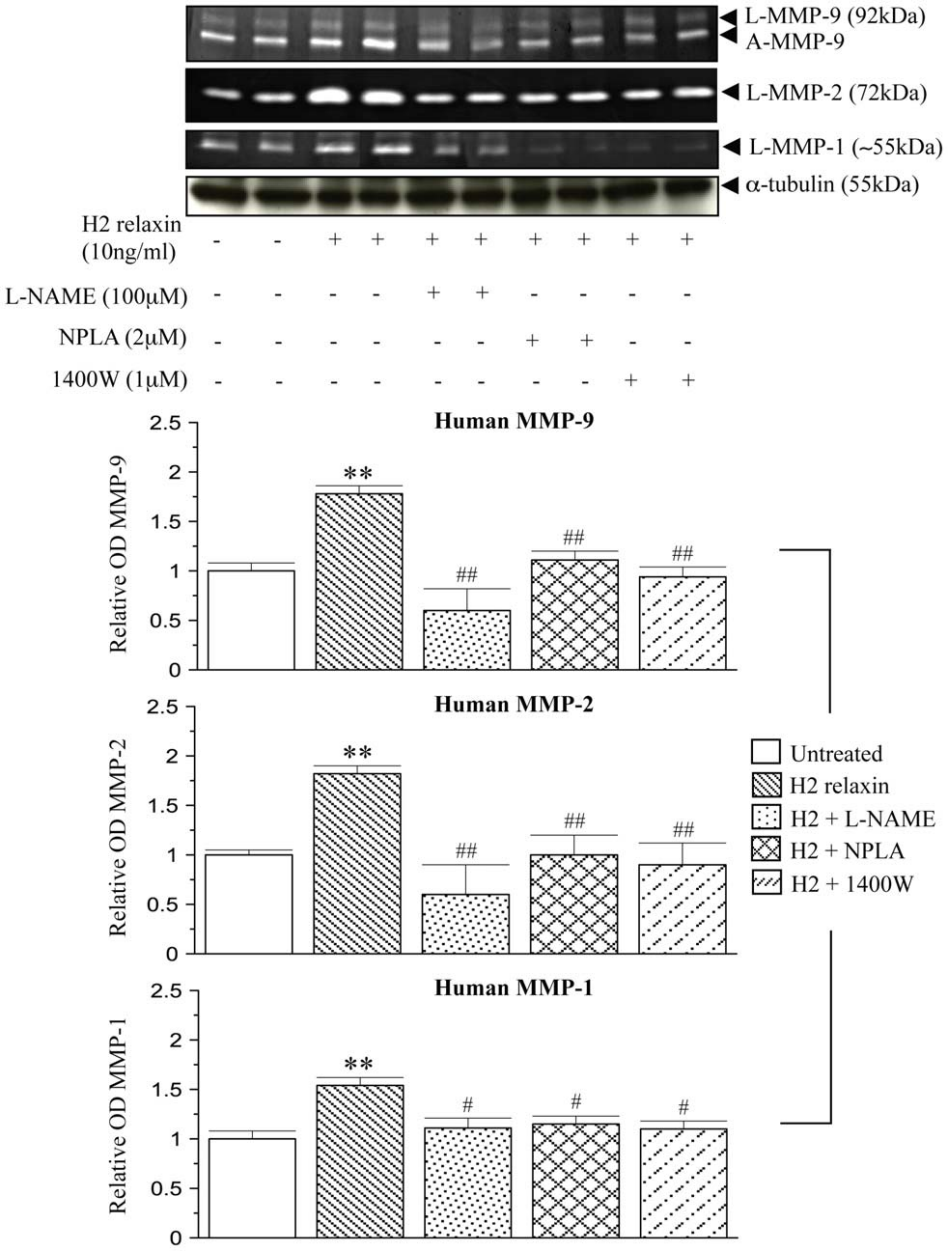
Relaxin signals through nNOS and iNOS to positively regulate MMPs in human myofibroblasts. Representative zymographs of L-MMP-9, A-MMP-9, L-MMP-2 and L-MMP-1 from TGF-β1-stimulated human dermal fibroblasts and cells treated with H2 relaxin (10 ng/ml) over 72 hours, in the absence or presence of the non-specific NOS inhibitor, L-NAME (100 µM); nNOS inhibitor, NPLA (2 µM); or iNOS inhibitor, 1400W (1 µM). Additional blots of α-tubulin demonstrate the quality and equivalent loading of protein samples. Also shown are the mean ± SE levels of each human MMP studied (which was derived from the latent and active forms), in the absence or presence of H2 relaxin and each inhibitor studied, as determined by densitometry scanning (from at least 3 separate experiments); and expressed as relative values to those of the TGF-β1 alone-treated group, which was expressed as 1 in each case. **p<0.01 vs TGF-β1 alone-treated cells; #p<0.05 and ##p<0.01 vs H2 relaxin alone-treated cells.

**Figure 3:**
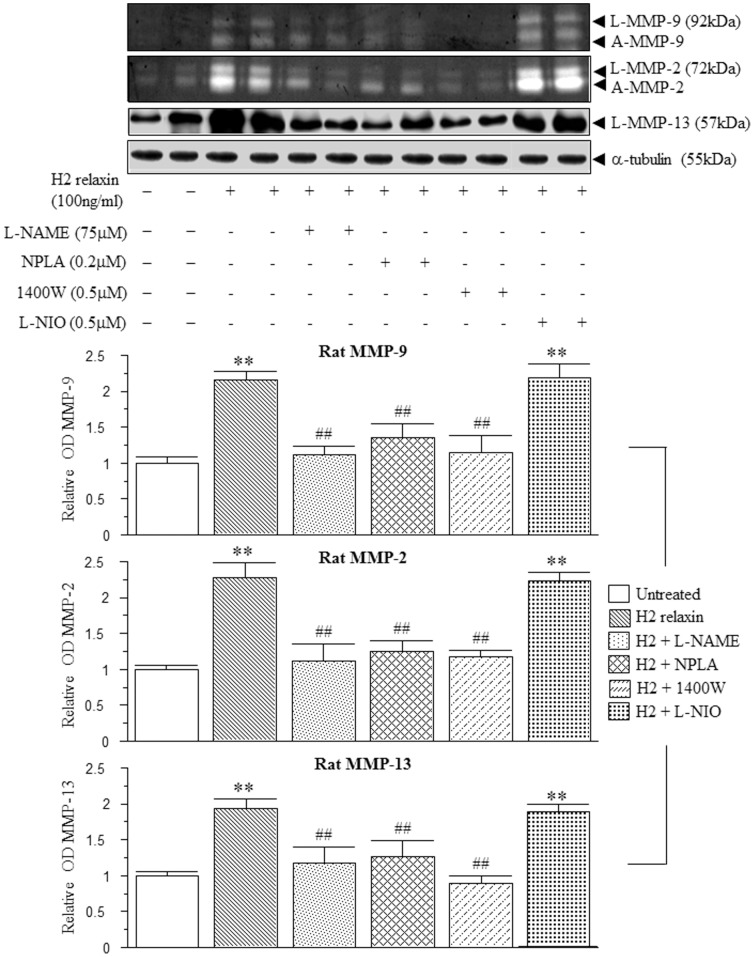
Relaxin signals through nNOS and iNOS to positively regulate MMPs in rat myofibroblasts. Representative zymographs of L-MMP-9, A-MMP-9, L-MMP-2, A-MMP-2 and Western blots of L-MMP-13 from untreated rat renal myofibroblasts and cells treated with H2 relaxin (100 ng/ml) over 72 hours, in the absence or presence of the general NOS inhibitor, L-NAME (75 µM); nNOS inhibitor, NPLA (0.2 µM); iNOS inhibitor, 1400W (0.5 µM); or eNOS inhibitor, L-NIO (0.5 µM). Additional blots of α-tubulin demonstrate the quality and equivalent loading of protein samples. Also shown are the mean ± SE levels of each rat MMP studied (which was derived from the latent and active forms), in the absence or presence of H2 relaxin and each inhibitor studied, as determined by densitometry scanning (from at least 3 separate experiments); and expressed as relative values to those of the untreated group, which was expressed as 1 in each case. **p<0.01 vs untreated cells; ##p<0.01 vs H2 relaxin alone-treated cells.

The MMP-promoting effects of H2 relaxin were almost completely, if not completely blocked by the presence of the general NOS inhibitor L-NAME (75–100 µM), the nNOS inhibitor NPLA (0.2–2 µM) and the iNOS inhibitor 1400W (0.5–1 µM) in both human (Figure 2) and rat (Figure 3) myofibroblasts (all p<0.05 vs H2 relaxin alone), but not the eNOS inhibitor L-NIO (0.5–5 µM) in the latter cells (Figure 3); while neither of these inhibitors affected MMP levels in the absence of relaxin (at the concentrations detailed above; data not shown).

**Figure 4:**
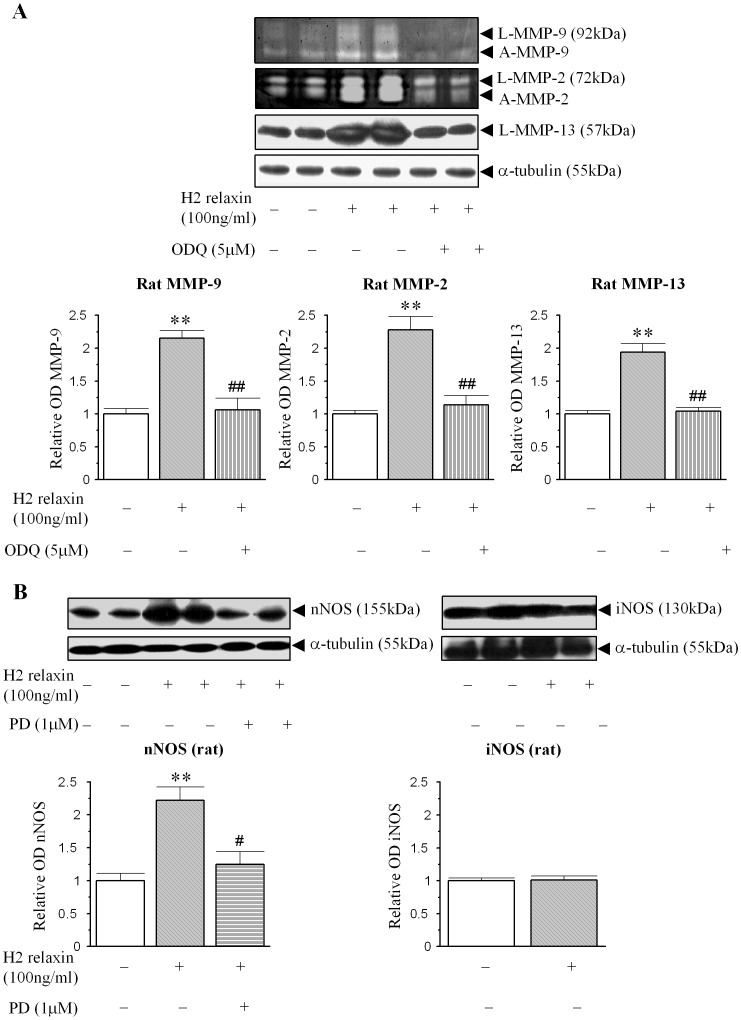
Relaxin initially signals through a pERK-nNOS-NO-cGMP-dependent pathway to up-regulate renal MMP levels. (A) Representative zymographs and Western blots of L-MMP-9, A-MMP-9, L-MMP-2, A-MMP-2 and L-MMP-13 from untreated rat renal myofibroblasts and cells treated with H2 relaxin ± the guanylyl cyclase inhibitor, ODQ (5 µM) over 72 hours. (B) Representative Western blots of nNOS and iNOS expression from rat renal myofibroblasts treated with H2 relaxin (100 ng/ml) over 72 hours; in the absence or presence of the ERK inhibitor, PD98059 (1 µM) for nNOS expression. Additional blots of α-tubulin (A, B) demonstrate the quality and equivalent loading of protein samples. Also shown are the mean ± SE levels of each MMP studied (which was derived from the latent and active forms) (A) in addition to nNOS and iNOS expression (B), as determined by densitometry scanning (from 4 separate experiments for each parameter studied); and expressed as relative values to those of the untreated group (A, B), respectively, which was expressed as 1 in each case. **p<0.01 vs untreated cells; #p<0.05 and ##p<0.01 vs H2 relaxin alone-treated cells.

Based on the strikingly similar pattern of the relaxin-induced increases in MMPs levels from human (Figure 1A, Figure 2) and rat (Figure 1B, Figure 3) myofibroblasts, the involvement of cGMP in the relaxin-mediated effects demonstrated; and further delineation of the involvement of nNOS vs iNOS to the effects of relaxin were further studied in rat renal myofibroblasts. The H2 relaxin-induced up-regulation of MMP-9, MMP-2 and MMP-13 levels was completely blocked by the presence of the guanylyl cyclase inhibitor ODQ (5 µM) (p<0.05 vs H2 relaxin alone; Figure 4A), confirming that relaxin was signalling through a NOS-NO-cGMP-dependent pathway to stimulate these MMPs; while ODQ alone did not affect MMP expression in the absence of relaxin. Furthermore, while our previous findings demonstrated that rat renal myofibroblasts expressed nNOS, but not eNOS [20], we additionally found that these cells expressed iNOS as well. The H2 relaxin-induced promotion of nNOS (Figure 4B), that was also demonstrated before [20], was found to be significantly inhibited in the presence of the ERK1/2 inhibitor, PD98059 (1 µM); suggesting that H2 relaxin's ability to stimulate ERK1/2 phosphorylation [20] was linked to its ability to up-regulate nNOS expression in myofibroblasts. On the other hand, H2 relaxin did not have any effects on iNOS expression, upon administration to these cells (Figure 4B); implying that relaxin was primarily signalling through a RXFP1-pERK-nNOS-NO-cGMP-dependent pathway to positively regulate MMPs; but that iNOS was somehow involved at a down-stream level.

### H2 relaxin signals through nNOS to positively regulate renal MMP expression and activity in vivo

**Figure 5:**
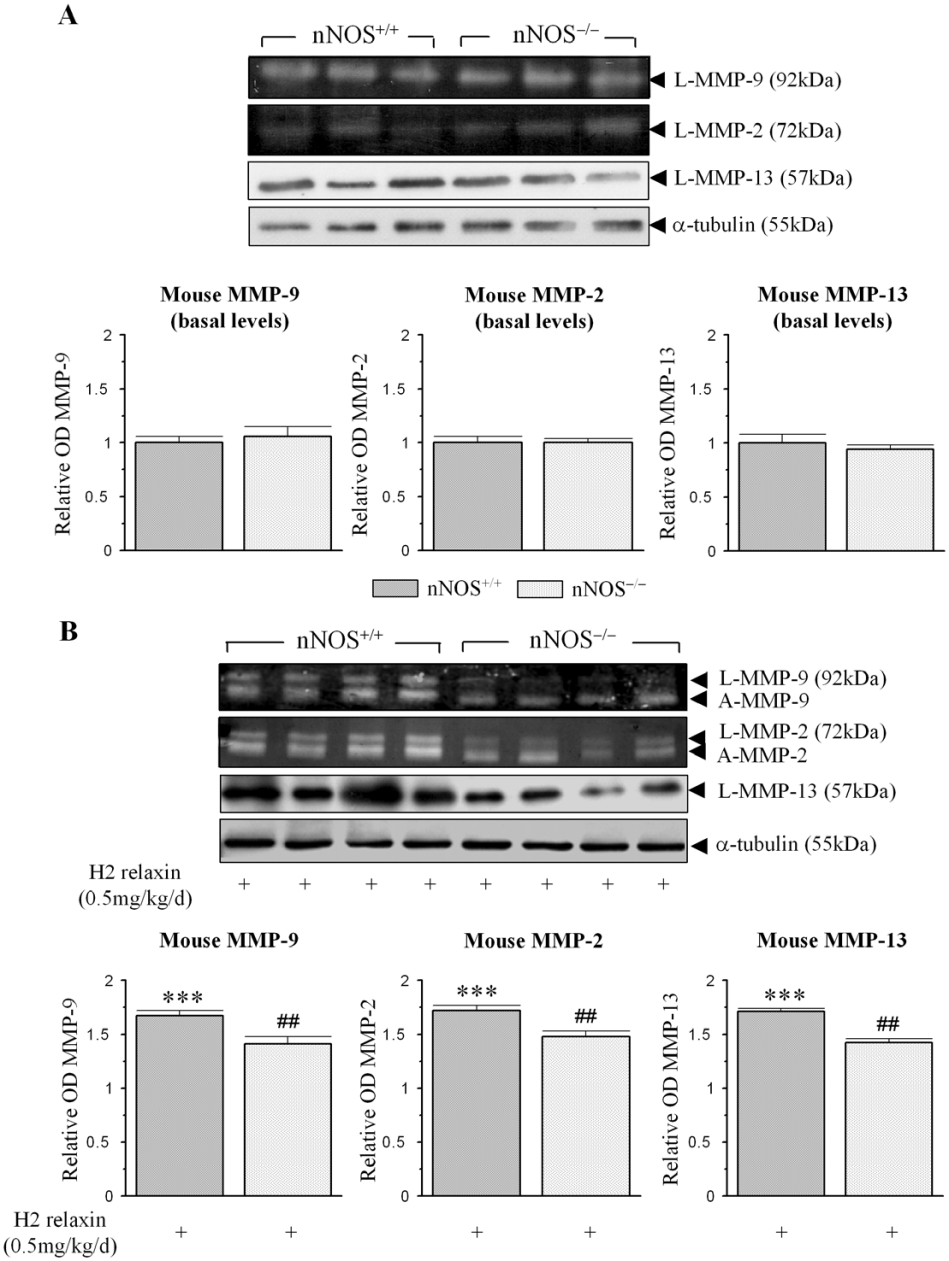
nNOS is required for the MMP-stimulating actions of relaxin in vivo. (A) Representative gelatin zymographs and Western blots of basal MMP-9, MMP-2 and MMP-13 levels from untreated nNOS^+/+^ and nNOS^−/−^ mice. (B) Representative zymographs and Western blots of L-MMP-9, A-MMP-9, L-MMP-2, A-MMP-2 and L-MMP-13 from kidney tissues of nNOS^+/+^ and nNOS^−/−^ mice, treated with H2 relaxin (0.5 mg/kg/day) for 7 days. Additional blots of α-tubulin (A,B) demonstrate the quality and equivalent loading of protein samples. Also shown are the mean ± SE levels of each MMP studied (A,B) (which was derived from the latent and active forms) from untreated vs H2 relaxin-treated animals; as determined by densitometry scanning (from the 3–4 samples per group analysed); and expressed relative to basal levels of each MMP analysed from the untreated nNOS^+/+^ group, which was expressed as 1 in each case. **p<0.01 vs corresponding basal levels from nNOS^+/+^ mice; #p<0.05 vs corresponding values from H2 relaxin-treated nNOS^+/+^ mice.

To confirm the involvement of nNOS in the H2 relaxin-mediated up-regulation of MMPs *in vivo* (as H2 relaxin has been demonstrated to stimulate MMPs in a number of tissues upon its administration to mice [18], [19], [42], [43]), renal MMP-2, MMP-9 and MMP-13 expression/activity was determined from untreated vs H2 relaxin-treated nNOS^+/+^ vs nNOS^−/−^ mice (Figure 5). Basal MMP-9, MMP-2 and MMP-13 levels were equivalent in untreated nNOS^+/+^ vs nNOS^−/−^ mice (Figure 5A). Compared to the levels of these MMPs found in H2 relaxin-treated nNOS^+/+^ mice (which were ∼67%, 72% and 71% higher than basal levels (all p<0.001 vs respective basal measurements; Figure 5B)), renal MMP-9, MMP-2 and MMP-13 expression/activity was found to be ∼40%, 33% and 41% lower (all p<0.01 vs levels in H2 relaxin-treated nNOS^+/+^ mice), respectively, in H2 relaxin-treated nNOS^−/−^ mice (Figure 5B); confirming that nNOS clearly played a role in the relaxin-induced stimulation of these MMPs.

### H2 relaxin abrogates TGF-β1-induced suppression of iNOS expression

**Figure 6:**
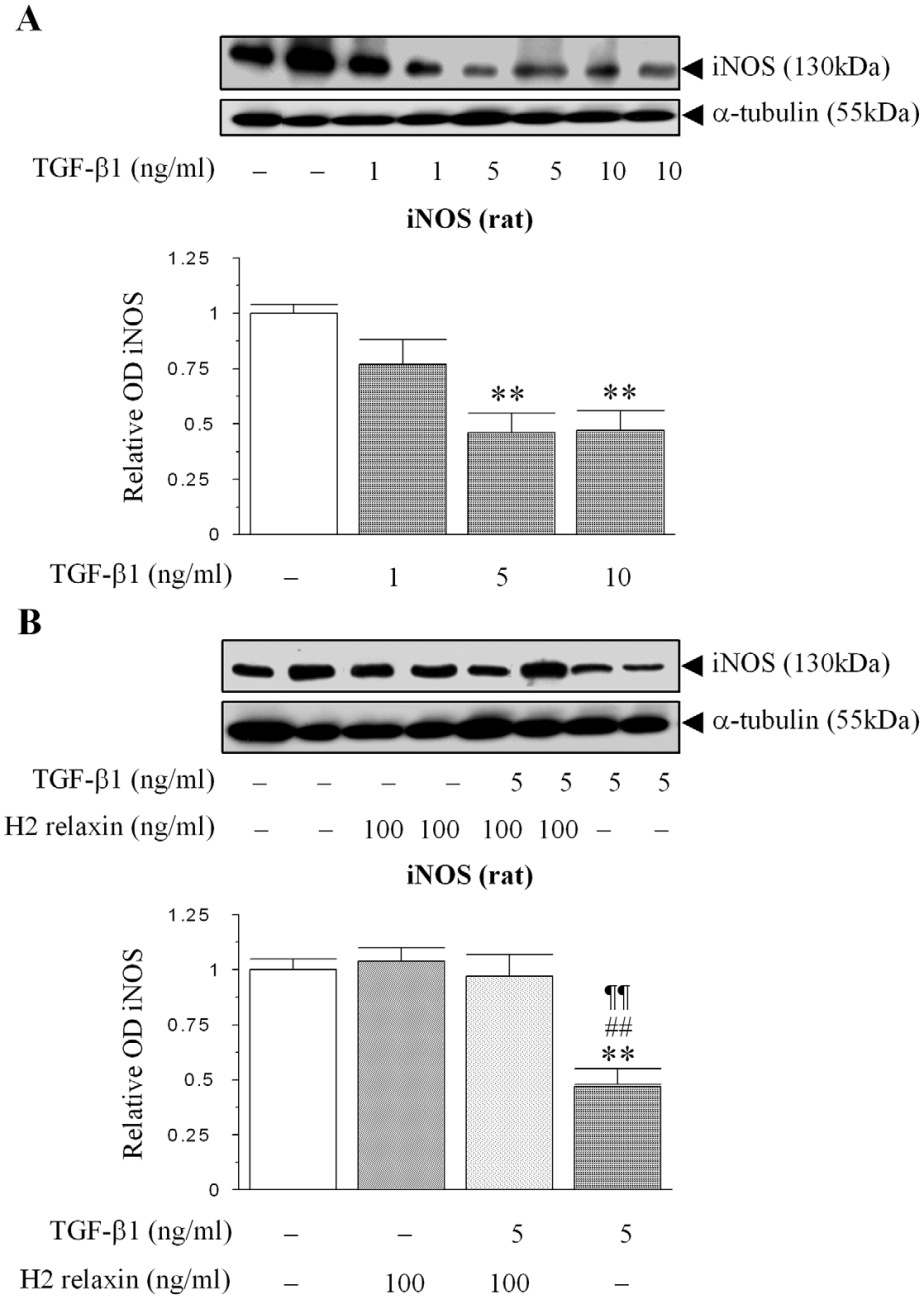
H2 relaxin abrogates TGF-β1-induced suppression of iNOS expression in renal myofibroblasts. (A) A representative Western blot of iNOS expression in response to increasing concentrations of exogenous TGF-β1 administration (1–10 ng/ml) to rat renal myofibroblasts. (B) A representative Western blot of iNOS expression in untreated, relaxin alone (100 ng/ml)-treated, relaxin (100 ng/ml) plus TGF-β1 (5 ng/ml)-treated and TGF-β1 (5 ng/ml) alone-treated renal myofibroblasts after 72 hours. Additional blots of α-tubulin (A,B) demonstrate the quality and equivalent loading of protein samples. Also shown are the mean ± SE levels of iNOS expression, as determined by densitometry scanning (from 3–5 separate experiments conducted in duplicate); and expressed as relative values to those of the untreated group, which was expressed as 1 in each case. **p<0.01 vs untreated cells; ##p<0.01 vs H2 relaxin alone-treated cells; ¶¶p<0.01 vs H2 relaxin+TGF-β1-treated cells.

To confirm that the involvement of iNOS (in the relaxin-mediated up-regulation of MMPs) was down-stream of H2 relaxin's ability to inhibit the Smad2/TGF-β1 axis, the effect of exogenous TGF-β1 (1–10 ng/ml) administration on iNOS expression from renal myofibroblasts was studied. TGF-β1 significantly reduced iNOS expression at concentrations of 5–10 ng/ml (Figure 6A), consistent with findings in other cell types studied [35]–[37]. This TGF-β1 (5 ng/ml)-induced suppression of iNOS expression was significantly abrogated in renal myofibroblasts co-treated with H2 relaxin (100 ng/ml) (Figure 6B); such that iNOS levels in H2 relaxin plus TGF-β1-treated cultures were equivalent to that measured in untreated and relaxin (100 ng/ml)-alone-treated cells (Figure 6B).

## Discussion

**Figure 7:**
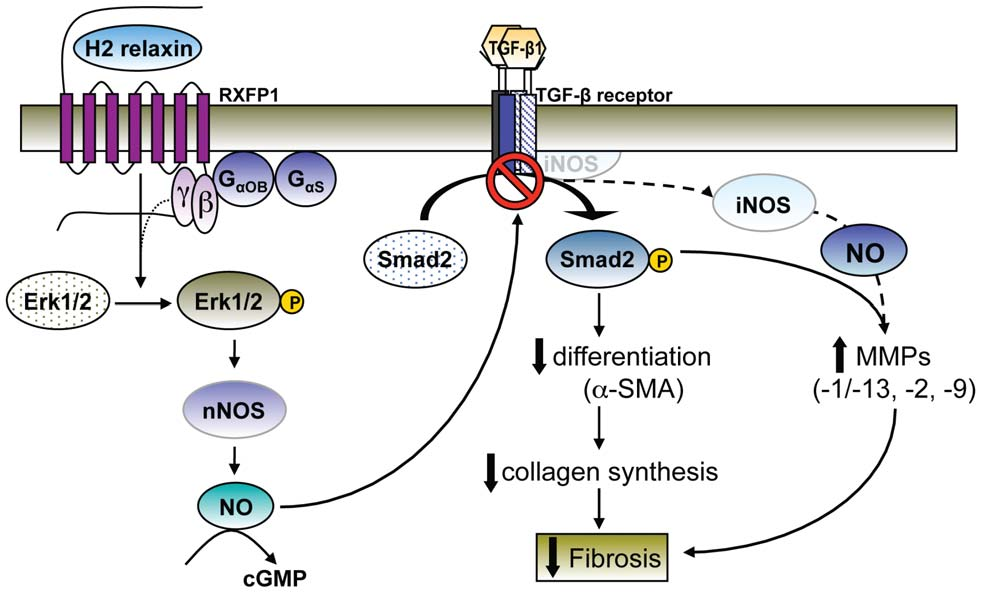
A schematic illustration of the proposed signal transduction mechanisms of H2 relaxin's anti-fibrotic actions, via the NO pathway. H2 relaxin binding to RXFP1 on myofibroblasts transiently stimulates Gαs and Gαob proteins; and ERK1/2 phosphorylation (pERK) over a longer period of time [20]. The H2 relaxin-induced stimulation of pERK is linked to its ability to signal through a nNOS-NO-cGMP-dependent pathway to inhibit Smad2 phosphorylation (pSmad2); which in turn, disrupts TGF-β1 activity and its down-stream effects on myofibroblast differentiation and myofibroblast-induced aberrant collagen production (which forms the basis of fibrosis). As TGF-β1 suppresses iNOS expression in a number of cell types including myofibroblasts, the H2 relaxin-induced activation of the RXFP1-pERK-nNOS-NO-cGMP-dependent pathway, releases iNOS, which through higher levels of NO, specifically contributes to the MMP-promoting actions of the anti-fibrotic hormone (associated with collagen degradation).

The matrix remodelling and anti-fibrotic actions of H2 relaxin are increasingly being recognised to be mediated via inhibition of the TGF-β1/pSmad2 axis [20]–[22]. More specifically, relaxin was found to signal through its primary receptor, RXFP1, Gαs and Gαob proteins, pERK and a nNOS-NO-cGMP-dependent pathway to inhibit pSmad2 as a means of interfering with the influence of TGF-β1 on renal myofibroblast differentiation and collagen production [20]. In this study, we demonstrate for the first time in TGF-β1-stimulated human dermal fibroblasts and rat renal myofibroblasts that express RXFP1, that relaxin primarily signals through this RXFP1-pERK-nNOS-NO-cGMP-dependent pathway to also up-regulate MMP-1 (and its rodent equivalent, MMP-13), MMP-2 and MMP-9; which are known to degrade collagens and other matrix proteins [26], [44]–[47]. Additionally though, we further demonstrated that iNOS is also specifically involved in the H2 relaxin-induced up-regulation of these MMPs, and that its up-regulation is a direct consequence of the H2 relaxin-mediated suppression of the TGF-β1/pSmad2 axis (Figure 7). These findings appear to be species and organ independent and confirm the central role of NO to the matrix remodelling and anti-fibrotic actions of relaxin.

Although the direct involvement of RXFP1 in the H2 relaxin-mediated effects on MMPs was not investigated in this study, our previous work has demonstrated that H2 relaxin specifically signals through its cognate receptor to mediate its matrix remodelling and anti-fibrotic actions. H2 and H3 relaxin (the latter which does not activate RXFP2; the cognate receptor for the related peptide, insulin-like peptide 3 (INSL3)) were both found to potently stimulate latent and active MMP-2 levels [38], while H2 relaxin also significantly increased MMP-13 levels [22] in rat cardiac ventricular fibroblasts that specifically expressed RXFP1 but not RXFP3 (the cognate receptor for H3 relaxin). Furthermore, both H2 and H3 relaxin were found to inhibit Smad2 phosphorylation, myofibroblast differentiation and collagen deposition in these cardiac fibroblasts [22]. In separate studies involving renal myofibroblasts, H2 relaxin could only prevent myofibroblast differentiation in cells isolated from the obstructed kidneys of RXFP1 wild-type mice, but not in corresponding cells isolated from RXFP1 knockout mice [20], confirming that the presence of RXFP1 was obligatory for relaxin to mediate these effects. Although, both RXFP1 and RXFP2 were also found to be present in the rat renal myofibroblasts used in this study [20]), INSL3 did not have any marked effects on renal myofibroblast differentiation, again suggesting that H2 relaxin most likely mediates its inhibitory actions on TGF-β1/pSmad2, myofibroblast differentiation and collagen production, while promoting MMPs via RXFP1.

While NO and cGMP appear to be key mediators involved in the matrix remodelling [20], [48], [49] and other actions [50], [51] of relaxin, it is likely that the NOS isoform associated with the effects of the hormone may vary depending on the specific action it carries out or the organ and/or cell type it targets. Clearly nNOS is involved in the collagen remodelling and anti-fibrotic actions of relaxin (in fibroblasts that it is constitutively expressed in), as the inhibition of nNOS significantly blocked the relaxin-induced down-regulation of pSmad2 and myofibroblast differentiation [20], as well as the relaxin-mediated up-regulation of MMPs; the latter being demonstrated in both human dermal and rat renal myofibroblasts. Although inhibitors to both nNOS and iNOS were able to block the MMP-promoting actions of H2 relaxin in both the human and rat myofibroblasts studied, our additional findings that i) H2 relaxin specifically increased nNOS expression without affecting iNOS expression in renal myofibroblasts; and ii) the inhibition of iNOS did not influence the ability of relaxin to down-regulate pSmad2 and myofibroblast differentiation (20), confirms the specificity of the inhibitors used; and that relaxin initially signals through a nNOS-NO-cGMP-dependent pathway to mediate its anti-fibrotic actions in general (whether it be via inhibition of pSmad2/TGF-β1, myofibroblast differentiation and collagen production or up-regulation of MMPs); and that cross-talk between nNOS and iNOS was likely not occurring up-stream of pSmad2/TGF-β1 inhibition, as a suggested mode of action by others [52]. Our added finding that ERK inhibition ameliorated the H2 relaxin-induced up-regulation of nNOS confirms that the hormone's ability to stimulate pERK [20] is linked to its ability to signal through NOSI. On the other hand, consistent with the absence of eNOS expression in the cells studied [20], the eNOS-specific inhibitor (L-NIO) did not affect the relaxin-mediated increase in MMP levels. Thus, eNOS did not appear to be involved in mediating the anti-fibrotic actions of relaxin, but appears to be involved in mediating the vasodilatory actions of the hormone [53]. Interestingly, although fibroblasts isolated from patients with idiopathic pulmonary fibrosis (IPL) constitutively expressed all NOS isoforms [49], relaxin was found to inhibit lung myofibroblast contractility via a iNOS-NO-cGMP and cGMP-dependent protein kinase G-mediated pathway; again confirming the central role that NO and cGMP play in facilitating the matrix remodelling actions of relaxin, but suggesting that the NOS isoform that is involved in relaxin's actions is perhaps dependent on the specific action being executed by the hormone. Thus, further work will be required to determine the extent to which relaxin mediates its various actions via the NOS isoforms available to it, even when multiple isoforms are present; and whether this can be manipulated to enhance its therapeutic potential.

A novel finding in this study was that unlike nNOS, iNOS was specifically involved in the MMP-promoting actions of H2 relaxin, independent of Smad2 phosphorylation and myofibroblast differentiation. This at first seems difficult to reconcile; both iNOS and nNOS produce NO and on face value we would expect NO to activate the same regulatory pathways. However, others have shown that the quantity of NO generated by iNOS is many magnitudes greater than that generated from constitutive isoforms (nNOS, eNOS) [54]. It seems plausible then that differential effects are concentration dependent, with iNOS generated-NO activating pathways different to those that are activated by nNOS generated-NO. Our observation that TGF-β1 suppresses iNOS expression is in agreement with observations in other cells [36], [37], including myofiboblasts [35], and provides important context. In these circumstances, a H2 relaxin-mediated inhibition of TGF-β1 signalling (via nNOS) removed an effective inhibitor of iNOS expression, which resulted in the corresponding iNOS-mediated increase in MMP expression and activity.

Although further work is required to fully understand the involvement of iNOS in the relaxin-mediated regulation of matrix components, it is interesting to note that other processes in which relaxin has been shown to mediate its actions through iNOS, including its ability to restore ileal motility in dystrophic mice [55] and promote vasodilatation [56], involve an up-regulation of MMPs: MMP-2 was recently shown to be essential for the growth and/or regeneration of tissues in dystrophic mice [57], while relaxin improves systemic and renal vasodilatation via actions on MMP-2 and MMP-9 [14], [58]. Additionally, as iNOS appears to be involved in promoting the actions of the MMPs studied in several other processes, including MMP-9 expression in patients with head and neck carcinomas [59]; MMP-2 levels in tissue repair process involving injured skeletal muscle [60]; and MMP-1, MMP-3 and MMP-13 activity in osteoarthritis cartilage turnover [61]; these combined findings suggest that the induction or release of iNOS is inherently linked to the induction of MMPs, independent of species and organ; while relaxin is able to activate the iNOS-NO-MMP interaction to mediate its anti-fibrotic actions.

From these studies, we conclude that H2 relaxin initially up-regulates MMP expression and activity via a RXFP1-pERK-nNOS-NO-cGMP-dependent pathway in human dermal and rat renal myofibroblsts. H2 relaxin was recently also shown to act through this pathway to inhibit the pSmad/TGF-β1 axis and thus, the influence of TGF-β1 on myofibroblast differentiation and collagen production [20]. The H2 relaxin-mediated suppression of TGF-β1 activity/signalling subsequently allows iNOS expression to be additionally involved in the MMP-inducing effects of the hormone (Figure 7). These findings confirm the central involvement of NO and cGMP to the matrix remodelling actions of relaxin, and suggest that manipulating the NOS isoforms available to relaxin may direct the specificity of its actions and its therapeutic potential.
